# Extensive hidden prophage diversity in Enterobacter species reveals host specificity and local distribution

**DOI:** 10.1099/mic.0.001660

**Published:** 2026-01-28

**Authors:** Danna Paola Bours-Lugo, Juan Manuel Hurtado-Ramírez, Armando Hernández-Mendoza, Ramón A. González, Adrian Ochoa-Leyva, Gamaliel López-Leal

**Affiliations:** 1 Laboratorio de Biología Computacional y Virómica Integrativa, Centro de Investigación en Dinámica Celular, Universidad Autónoma del Estado de Morelos, Cuernavaca, Mexico; 2 Instituto de Biotecnología, Universidad Nacional Autónoma de México, Cuernavaca, Morelos, Mexico; 3 Centro de Investigación en Dinámica Celular (CIDC), Universidad Autónoma del Estado de Morelos, Cuernavaca, Mexico; 4 Departamento de Microbiología Molecular, Instituto de Biotecnología, Universidad Nacional Autónoma de México, Cuernavaca, Mexico

**Keywords:** bacteriophages, *Enterobacter*, phage–host interactions, prophage diversity, prophages

## Abstract

Bacteriophages are key drivers of bacterial evolution, particularly through their integration as prophages within host genomes. However, the diversity and host specificity of prophages in relevant pathogens such as *Enterobacter* species remain poorly characterized. In this study, we revealed the diversity of prophages, mapped their distribution and explored their relationships with their bacterial hosts. We analysed 3,661 prophage sequences identified from the genomes of 20 different *Enterobacter* species. This analysis uncovered an extensive hidden diversity, comprising 1,617 phage genera and 2,423 phage species – nearly 80% of which were singletons – highlighting an exceptionally rich prophage landscape. We found substantial variation in prophage species richness across host species and isolation sources, with *Enterobacter kobei* and environmental isolates exhibiting the highest richness. Prophage populations showed strong host specificity and limited cross-species transmission. Moreover, prophages exhibited geographic structuring and significant congruence between host and prophage phylogenies, as well as with the ecological lifestyles of their bacterial hosts. Although we found phages of the same species infecting different host species, these events were infrequent. Finally, bacterial genomes encoded diverse defence systems, mainly PDC-S07, RM type I–II and gabija, whereas only 8.9% of prophages encoded anti-defence systems, mostly anti-CBASS and anti-RM. Overall, this study provides new insights into the diversity of *Enterobacter* prophages and underscores their ecological and clinical relevance in shaping host adaptation and phage–host dynamics.

**Impact Statement**

*Enterobacter* species are emerging opportunistic pathogens increasingly implicated in hospital-acquired infections. Although prophages play a pivotal role in bacterial genome evolution and host adaptation, their diversity and distribution across *Enterobacter* species remain largely uncharacterized. In this study, we performed a large-scale genomic analysis of 3,661 prophages from 20 *Enterobacter* species and uncovered an extensive and previously hidden prophage diversity. Our analysis revealed significant differences in prophage species richness across both host species and isolation sources, with *Enterobacter kobei* and environmental isolates exhibiting the highest richness. Prophage populations were strongly structured by host species and geography, showing limited cross-species transmission and a high degree of congruence between phage and host phylogenies. These findings highlight the structured and lineage-specific nature of prophage populations in *Enterobacter* and provide valuable insights into phage–host coevolution and microbial biogeography.

## Data Summary

All bacterial genomes were obtained from the National Center for Biotechnology Information, GenBank/RefSeq (October 2024, accessions listed in Table S1). Validated prophage sequences and isolate bacteriophage sequences, and associated metadata, are publicly available at GitHub (https://github.com/BCVI/Enterobacter_prophages) and archived in Zenodo under DOI: 10.5281/zenodo.16989452. No new raw sequencing data were generated.

## Introduction

Viruses are the most abundant biological entities on Earth [1]. Within this vast virosphere, bacteriophages (phages) infect only prokaryotic microorganisms [2]. Phages can replicate through a lytic cycle (as in virulent phages), integrate into the host genome as prophages or persist as extrachromosomal plasmid-like elements [3]. Once integrated, prophages replicate alongside the host chromosome and transmit vertically from the original infected cell to its progeny during cell division [4]. These genetic elements play an important role in bacterial evolution and ecology. They drive horizontal gene transfer and genetic diversification, enhancing the adaptive potential of their hosts [5,6]. By introducing new genetic material and mediating gene exchange among populations [7,8], they shape bacterial pathogenicity, resistance and ecological fitness [7,10]. Additionally, prophages can block infection by other phages that use the same receptor, providing superinfection immunity [9,10]. Also, the presence of orthologous prophages enables recombination with virulent phages that could potentially generate defective particles, active dormant prophages [11], or occasionally, produce novel infective viral variants [12,13]. On the other hand, the evolutionary interplay between phages and their bacterial hosts represents a continuous arms race [14]. Bacteria can encode diverse antiviral defence systems against phage infection, while phages develop strategies to evade these systems [15]. Notably, several prophages have been shown to encode anti-defence systems that suppress host immunity. These mechanisms could contribute to prophage persistence and stability within bacterial genomes [16]. Nevertheless, despite the development of computational tools to identify defence and anti-defence systems, the extensive diversity and rapid evolution of these protein families continue to hinder the reliable detection of homologous anti-defence genes in prokaryotic and phage genomes [15].

Importantly, many relevant pathogens carry abundant prophages in their genomes [17]. Advances in nucleotide sequencing and bioinformatics have revealed that mobile genetic elements, including prophages, constitute a substantial portion of bacterial genomes [18]. For example, in previous studies, we mined 13,713 complete bacterial genomes and found that 75% harboured identifiable prophages, with multiple bacterial genera showing an average of more than five prophages per genome – and at least 1 genome harbouring more than 20 [17]. Moreover, species within the genera *Enterobacter*, *Acinetobacter* and *Pseudomonas* show significantly higher prophage enrichment in pathogenic isolates compared to non-pathogenic counterparts [17]. While prophage populations have been relatively well characterized in *Acinetobacter* and *Pseudomonas* species [19,21], studies addressing prophage diversity across *Enterobacter* species remain largely lacking.

Species within the genus *Enterobacter* occupy diverse ecological niches within the *Enterobacteriaceae* family, including clinical, environmental and animal-associated habitats [22]. Moreover, several *Enterobacter* species act as plant pathogens [23] and also belong to the human gut commensal microbiota [24]. Some species, such as *Enterobacter cloacae*, *Enterobacter asburiae* and *Enterobacter hormaechei*, are frequent causes of hospital-acquired infections in humans [25]. Due to their clinical impact and rising antibiotic resistance, the World Health Organization has classified *Enterobacter* spp. as critical priority pathogens [26]. Across the Americas, nearly 30–50% of *Enterobacter* isolates exhibit resistance to third-generation cephalosporins, a proportion that continues to rise [26]. Consequently, alternative strategies such as phage therapy may offer promising options against these superbugs. However, to ensure the proper development of this strategy, it is important to understand and characterize the prophage populations in relevant pathogens (such as *Enterobacter* species) to elucidate phage–host dynamics [27].

To date, no comprehensive studies have focused on profiling prophage populations across the entire *Enterobacter* genus. Existing work has targeted only a few species, such as *E. cloacae* [7], *E. asburiae* [28] and *E. hormaechei* [29], but these analyses involved limited genome samples. On the other hand, only 117 genomes of phages infecting *Enterobacter* species had been deposited in the National Center for Biotechnology Information (NCBI) (accessed October 2024). However, from this perspective, scientists have preferentially sequenced more bacterial than viral genomes (at least in the context of *Enterobacter* species), thereby unintentionally capturing a significant portion of their phages (in lysogenic state). As such, exploring prophage diversity represents a valuable resource for understanding phage–host dynamics. Recent advances in sequencing technologies and bioinformatics have greatly expanded our ability to explore the remarkable diversity of prophages [18]. However, most available tools are limited to assessing prophage completeness and genomic quality. A major limitation remains the uncertainty in determining whether these elements are potentially inducible. Consequently, identifying active prophages continues to be a bioinformatic challenge, often requiring complementary validation through traditional microbiological approaches.

In this study, we investigate phage and prophage diversity across multiple *Enterobacter* species. We analysed a total of 3,778 bacteriophage sequences using comparative genomics and phylogenomics to deliver a comprehensive characterization of phage diversity and elucidate viral–host relationships.

## Methods

### Genomes used and prophage identification

To conduct our study, 757 complete bacterial genomes of the *Enterobacter* genus were downloaded from NCBI in October 2024. The quality of the genomes, namely, completeness and contamination percentages, was determined using CheckM2 (v.1.0.1) [30]. Only complete and uncontaminated genomes were included (completeness ≥95% and contamination ≤5%) and are listed in Table S1 (available in the online Supplementary Material). Biosample information for all genomes was obtained using efetch from E-utilities [31]. We collected the metadata for the following sections: isolation source, isolation site, host, environmental medium and sample type. We then categorized the isolates as human, animal, clinical (this category corresponds to host isolates coming from instrumentation and equipment) and environmental. When this information was not available, it was grouped in the N/A category. Prophage predictions were carried out by VirSorter2 (v.2.2.4) [32], using the following parameters: --min-length 5000 --min-score 0.5 --include-groups dsDNAphage. The quality of the prophage genomes was checked using CheckV (v.1.0.3) [33]. Only prophage sequences assigned as high-quality or complete by checkv-quality were considered for downstream analysis. We followed the publicly available protocol for the validation of the first-instance prophage prediction (dx.doi.org/10.17504/protocols.io.bwm5pc86). Briefly, the final quality prophages analysed by CheckV were validated in a second screening using VirSorter2. All genomes were annotated using prokka (v1.14.6) [34] and pharokka (v1.7.5) [35] for bacterial and viral genomes, respectively. In Pharokka, we used the following dependencies: Prodigal v2.6.3 [36] for gene prediction and MMseq2 (v 1.4.0) [37] to assign functional annotations against the PHROGs database [38]. Pharokka also compares predicted proteins against curated reference databases of bacterial virulence factors (VFDB) [39] and antimicrobial resistance genes (CARD) [40]. A second screening was carried out using the Resistance Gene Identifier (RGI) model to detect antibiotic-resistant genes (ARGs), which is based on protein homology and SNP detection, implemented in the Comprehensive Antibiotic Resistance Database (CARD) tool [40].

All bacteriophage sequences analysed during this study are available in the GitHub repository (https://github.com/BCVI/Enterobacter_prophages) and permanently archived in Zenodo (DOI: 10.5281/zenodo.16989452).

### Detection of bacterial defence and prophage anti-defence systems

To identify bacterial defence systems, we analysed 747 *Enterobacter* genomes using PADLOC (v2.0.0) [41]. System presence was determined according to published criteria [42]. For systems not previously described, a defence system was considered present when at least two proteins belonging to the same system were detected within a genome. To identify anti-defence systems in prophages, we used DefenseFinder (v2.0.1) [42] with the --antidefensefinder-only option, which restricts detection to known anti-defence genes.

### Phylogenetic reconstruction of the genus *Enterobacter*

We aimed to construct homologous groups from *Enterobacter* bacterial genomes to gain insights into their evolutionary relationships. First, we ran Roary [43], setting the blast search parameters to a length coverage of ≥80% and an amino acid sequence identity of ≥80%. We created homologous groups with only one copy gene per genome, which we referred to as single-gene families (SGFs). SGFs were considered for the phylogenetic analysis. The SGFs were aligned with muscle (v3.8) [44], specifying 50 iterations. Then, to create a DNA alignment in frame, we used the program TRANALING [45], which is part of the EMBOSS suite (v6.6.0). Additionally, we discarded SGFs with recombination signals using PhiPack (v1.1.0) [46] with a *P*-value cutoff of 0.05. Then, the SGFs that did not show recombination signals were concatenated to form a super-alignment. Based on this alignment, we constructed a maximum likelihood (ML) phylogeny, selecting (TIM+F+I+G4) as the best model suggested by IQ-TREE 2 (v2.0.7) [47]. We ran a non-parametric bootstrap analysis (100 replicates) on the ML phylogeny to establish the support for the clades. *Lelliottia nimipressuralis* (GCA_013898335) and *Lelliottia amnigena* (GCA_000016325) were used to give directionality to the tree [48]. We assessed the genomic relatedness among isolates using average nucleotide identity (ANI) analysis implemented in pyani with the MUMmer algorithm (ANIm) [49]. Genome pairs sharing ≥95% identity were considered to belong to the same species [50]. The resulting ANI matrix was visualized using the pheatmap (v1.0.12) package in R.

### Bacteriophage clustering at the genus and species levels and phylogenetic reconstruction

To determine bacteriophage diversity, we first defined our prophage populations into species and genera following ICTV (International Committee on Taxonomy of Viruses) standards using vClust (v1.3.0) [51]. Briefly, we performed an analysis similar to VIRIDIC [52] by calculating the total average nucleotide identity (tANI) between prophage genomes. Based on the tANI values, we classified viruses into species (≥95% tANI) and genera (≥70% tANI). We then constructed a neighbour-joining tree using the R ape (v5.6.1) and ggtree (v3.0.4) packages based on the pairwise identity matrix using only prophages that clustered into a genus with more than ≥5 members (to build a more robust and decisive tree). Similarly, the prophage species with ≥7 members were visualized as a tANI (≥95%) network using Cytoscape v.3.8 [53]. Bacteriophage taxonomic classification was carried out using taxMyPhage (v0.3.4) [54].

### Congruence between phylogenetic trees

To assess cophylogenetic patterns between *Enterobacter* hosts and their prophages, we conducted two complementary global-fit analyses: Procrustes Approach to Cophylogeny (PACo) [55] and ParaFit [56]. First, we inferred host and prophage phylogenies independently (see above). These phylogenies were converted into pairwise patristic distance matrices and the host–phage associations were encoded as a binary matrix. The PACo analysis was performed using the paco package in R (v 0.4.2). The model was fitted using the default principal coordinate decomposition of distance matrices and Procrustean superimposition. Statistical significance of the global fit was evaluated using 10,000 permutations. ParaFit analysis was carried out using the vegan package in R (v2.6.4), with significance assessed via 999 permutations of the host–phage association matrix. To examine individual associations under the PACo framework, we computed squared residuals from the Procrustes superimposition. Associations with residual values above the 95th percentile threshold were classified as potential host-switching events, while associations below this cutoff were considered consistent with codivergence.

### Prophage diversity and geographical distribution

We evaluated the relationship between phage species or genera and both geographic origin, isolation source and host species. To do this, we calculated the uncertainty coefficient (UC), using the R DescTools v0.99.44, between prophage (phage genera and species levels) and host features such as geographic locality, isolation source and host species (*Enterobacter* species).

### Rarefaction analysis of prophage species richness

To compare prophage species richness while controlling for differences in sample depth, we performed rarefaction analyses using the vegan package in R (v2.6.4). Community matrices were constructed by aggregating prophage species counts per *Enterobacter* host species and per isolation source. For isolation sources, we included all categories and rarefied richness to 102 sequences, corresponding to 75% of the total sequences in the least-sampled source (clinical). For host species, only those with ≥50 total sequences (prophages) were retained, and richness was rarefied to 63 sequences, corresponding to 75% of the total sequences in the least-sampled host (*Enterobacter ludwigii*). Using 75% of the total sequences in the least-sampled group as the rarefaction depth ensured that all categories were included in the analysis while minimizing the loss of diversity information from more deeply sampled groups. Rarefaction curves were generated using a fixed subsampling depth for all groups, and normalized richness values were extracted from the curves to enable direct comparison across categories.

### Statistical analysis and other tools used

Statistical analyses were conducted in R (v4.3.2). Differences in the number of prophages according to host isolation source and *Enterobacter* species were evaluated using the Wilcoxon test (packages ggplot2 and dplyr). Pairwise comparisons of the number of prophages across *Enterobacter* species were evaluated using Mann–Whitney U tests with Holm–Bonferroni correction. Paperpal was used to improve the clarity and grammar of the English language.

## Results

### High abundance of prophage across *Enterobacter* species

To explore prophage populations across different *Enterobacter* species, we analysed only high-quality and complete bacterial genomes (see the ‘Methods’ section). We kept 747 genomes representing 20 distinct species, according to NCBI’s taxonomic classification (Table S1). Additionally, genomes were collected from 35 different countries. Of these, China (21.41%), the USA (12.44%) and Japan (11.64%) showed the highest representation.

**Fig. 1.**
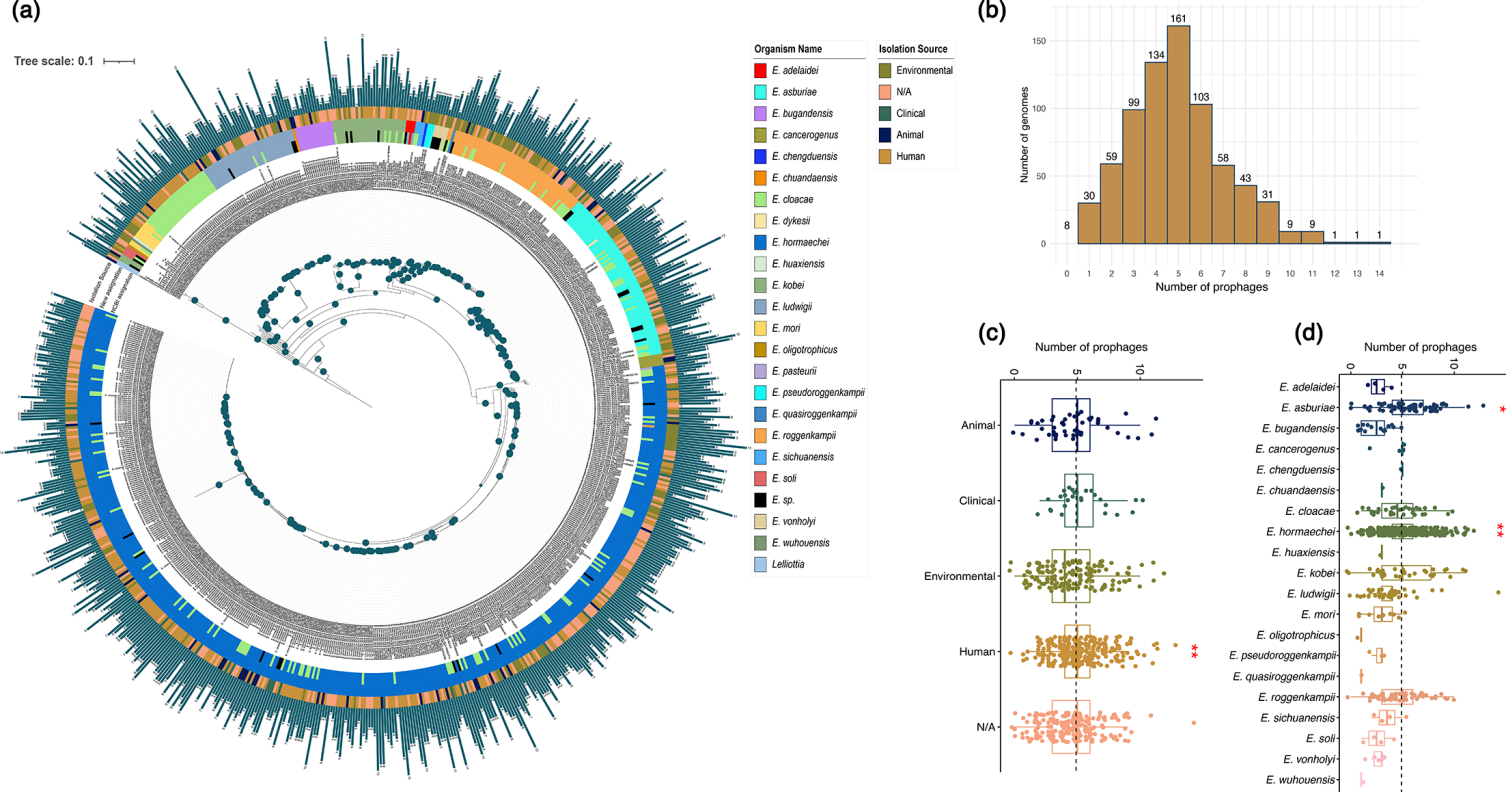
*Enterobacter* species phylogeny and prophage distribution. (**a**) ML phylogeny shows the relationships among all the genomes. The three outer circles represent (inner to outer): the NCBI species assignment, the species assignment inferred from the phylogeny and the isolation source (when available in the metadata; see the ‘Methods’ section). Colour codes for *Enterobacter* species and isolation sources are shown in the panels. Bar plots next to the circles denote the number of prophages identified for each genome. The species *L. amnigena* (GCA_000016325) and *L. nimipressuralis* (GCA_013898335), shown in lightsteelblue, were used as an outgroup to root the phylogeny. The tree scale is the number of substitutions per site, and bootstrap values higher than or equal to 95 are depicted with blue circles at the internal nodes of the phylogeny. (**b**) Histogram depicting the distribution of total prophages in *Enterobacter* genomes. Box plot representations of the number of prophages according to host isolation source (**c**) and *Enterobacter* species (**d**). Red asterisks indicate significant differences based on the Wilcoxon test (*P-value ≤0.05; **P-value ≤0.01). The dotted line indicates the overall average number of prophages among all *Enterobacter* genomes.

To achieve more accurate species identification – given the complex taxonomy of *Enterobacter* species – we reconstructed an ML phylogeny using 24 SGFs and without recombination signals (see the ‘‘Methods’ section). We first observed that most NCBI-assigned species clustered into well-supported clades (≥95% bootstrap support). Nevertheless, we reassigned 20.74% (155 genomes; Fig. 1a, second ring from the inside out) of the genomes based on the ML phylogeny (Fig. 1a). For example, those genomes previously labelled by NCBI as *Enterobacter* spp. (Fig. 1a, black labels), *Enterobacter roggenkampii* (species panel, orange labels) and *E. cloacae* (grass green labels) grouped within a major, well-supported clade corresponding to *E. hormaechei* (Fig. 1a, blue labels). We, therefore, considered these genomes as *E. hormaechei* for all downstream analyses and so on for all observed cases (Table S2). The species assignation recovered from the phylogenetic analysis was validated through an ANI comparison (Fig. S1). Genomes forming monophyletic clades in the species tree exhibited ANI values ≥95%, the established threshold for species designation [57,58]. Cases of misclassification were also supported by their corresponding ANI values. Overall, our analysis yielded a robust phylogeny for *Enterobacter* species and revealed several misclassified genomes. Moreover, the ML phylogeny was consistent with previous studies [59]. Finally, the most abundant species in our dataset were *E. hormaechei* (59.70%), *E. asburiae* (9.90%), *E. roggenkampii* (8.29%), *E. ludwigii* (5.48%), *E. kobei* (4.55%) and *E. cloacae* (4.28%).

Interestingly, we identified a total of 3,661 high-quality prophages (see the ‘Methods’ section), with a mode and mean of 5 and 4.9 prophages per genome, respectively. Prophages were present in all species analysed, with 98.92% of genomes harbouring at least one prophage. Remarkably, 39.49% of genomes contained between four and five prophages (Fig. 1b). We then assessed whether prophage abundance was associated with the isolation source and/or *Enterobacter* species. Genomes isolated from human sources exhibited a higher number of prophages in contrast with other isolation sources (Fig. 1c). Additionally, genomes belonging to *E. hormaechei* (*P*-value≤0.01) and *E. asburiae* (*P*-value≤0.05) were also enriched in prophages (Fig. 1d). However, as noted above, more than 50% of our dataset corresponds to *E. hormaechei*, which could bias the statistical outcomes. To address this, we first assessed whether there were overall differences in prophage abundance among *Enterobacter* species. We applied a Kruskal–Wallis test (*H*=89.44, *P*<0.0001), which indicated that at least one species differed significantly from another in prophage abundance, meaning that prophage numbers were not homogeneous across species. We then conducted pairwise comparisons using two-tailed Mann–Whitney *U* tests with Holm–Bonferroni correction for multiple testing. The results showed that *E. asburiae*, *E. bugandensis* and *E. hormaechei* harboured significantly higher numbers of prophages compared with other *Enterobacter* species (Fig. S2).

### Extensive hidden prophage diversity in *Enterobacter* species

To explore the diversity of *Enterobacter* prophages, we first contextualized our dataset by comparing the prophages identified in this study with 117 previously reported *Enterobacter* phage genomes retrieved from NCBI. We then defined phage species and genera by calculating pairwise genomic similarities among all sequences (see the ‘Methods’ section). Interestingly, we identified 2,423 phage species, of which 79.98% were singletons (1,938 prophages), indicating these phage species were unique to individual genomes. *E. hormaechei* and *E. asburiae* were the species with the highest number of singleton prophages. However, given the uneven representation of *Enterobacter* species in our dataset, we normalized the number of singletons per species by dividing the number of singletons by the number of genomes analysed for that species. *E. kobei* exhibited the highest singleton density (4.47 singletons per genome), although most species displayed densities ranging between 3 and 4 (Fig. S3).

**Fig. 2.**
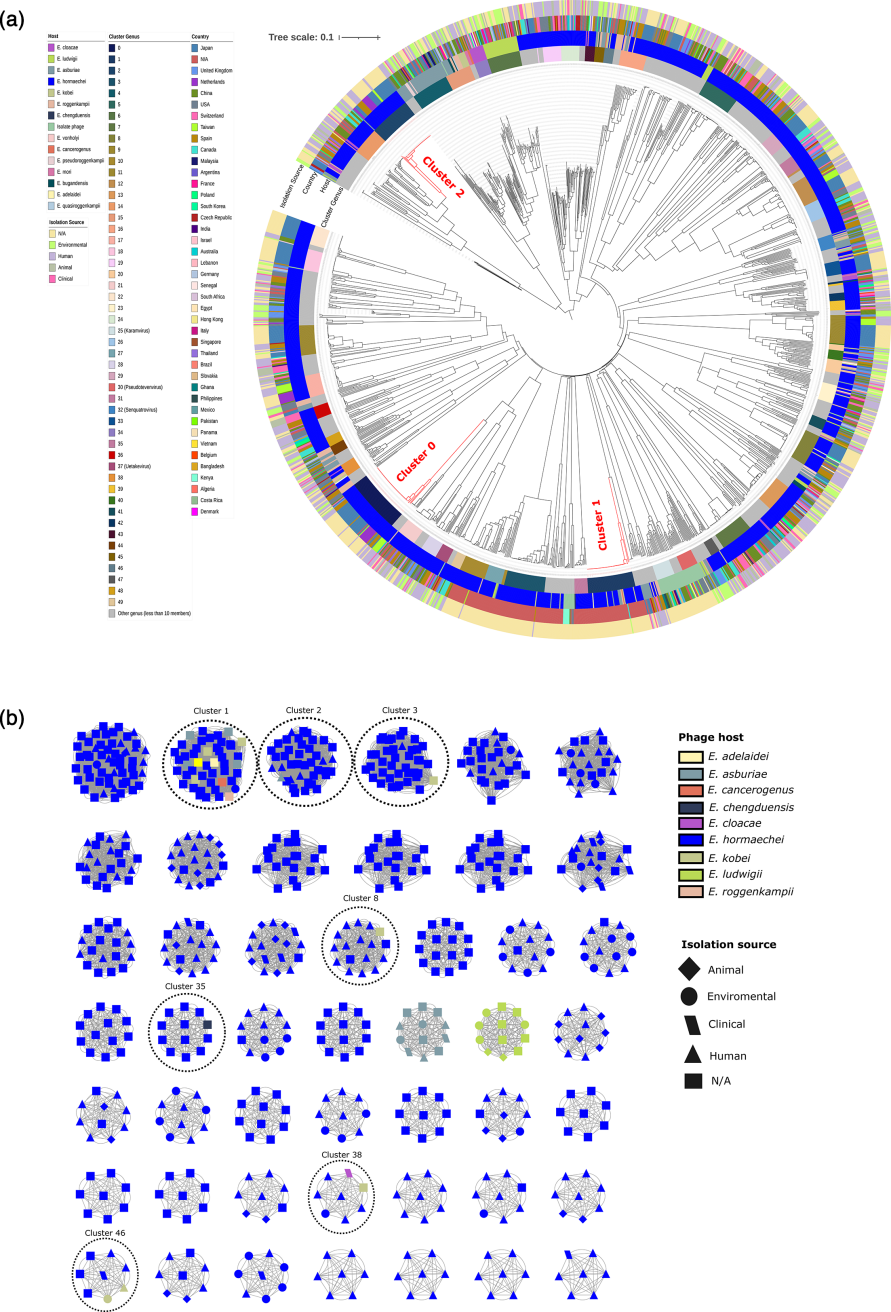
Phylogeny and network analysis of *Enterobacter* phages and prophages. (**a**) The neighbour-joining phylogenetic tree was constructed using 1,710 phage genomes, including only genera with more than 5 members, based on an intergenomic similarity matrix calculated with vClust at the genus level (≥70% tANI; see the ‘Methods’ section). For clarity, only genera with ten or more members are shown in colour (see cluster genus panel). The outer rings indicate the host isolation source, *Enterobacter* species and country of origin, obtained from the genome metadata where each prophage was identified (see the ‘Methods’ section). Clades corresponding to the three most prevalent genera are highlighted in red. (**b**) Network representation of *Enterobacter* bacteriophages at the species level, for the sake of clarity, only species having seven or more sequences are displayed (see the ‘Methods’ section). Nodes are colour-coded according to the *Enterobacter* species, and node shapes indicate the host isolation source. Dashed circles highlight the presence of prophages of the same species derived from different host species.

At the genus level, we identified 1617 phage genera. Next, to build a robust tree, singletons were excluded [60], and only genera comprising five or more members were used to construct a neighbour-joining phylogeny (see the ‘Methods’ section), which revealed a high degree of genetic diversity among prophages (Fig. 2a). Many genera are represented by only a few members, indicating that most prophage genera are sparsely distributed across *Enterobacter* genomes. The most prevalent phage genera were cluster 0, cluster 1 and cluster 2, with 57, 49 and 43 phages, respectively. Remarkably, members within these genera were nearly identical, as reflected by their extremely short branch lengths (Fig. 2a; red branches). Except for the members of cluster 0, which were found exclusively in *E. hormaechei*, the prophages of other major clusters (clusters 1 and 2) were distributed across multiple host species. These highly conserved prophages were also found in genomes from different countries and isolation sources. In other words, only a few phage species were associated with more than one host species. Notably, prophages belonging to cluster 1 were identified in six distinct *Enterobacter* species (Fig. 2b). Additionally, prophages from clusters 2, 3, 8, 35, 38 and 46 were also found in more than one *Enterobacter* species (Fig. 2b; see dashed circles). However, these events were less frequent compared to cluster 1. Furthermore, clustering analysis revealed that most prophage species group according to the isolation source of their bacterial hosts. However, some prophage species were identified in hosts from different sources. The most noteworthy prophage species are those detected in human isolates (Fig. 2b, triangles) and animal isolates (Fig. 2b, diamonds). In addition, prophage species occurring across different isolation sources were relatively uncommon. Most phage species were exclusive to a single isolation source, with only a few shared among different sources. The most notable overlap was observed between human-associated and environmental isolates, which shared 31 phage species in total (Fig. S4).

**Fig. 3.**
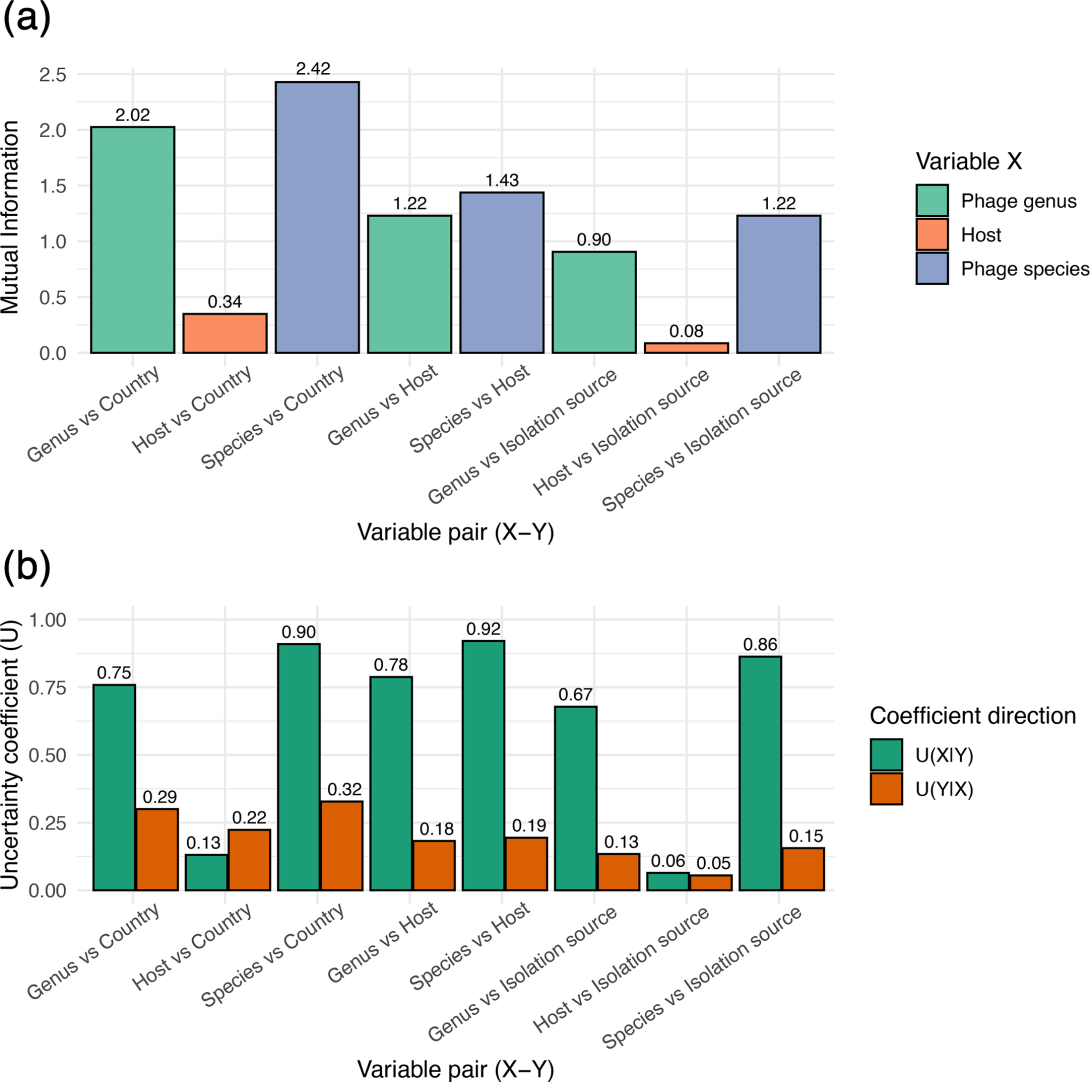
Phage–host associations measured by mutual information and uncertainty coefficients. (**a**) Mutual information between phage and host metadata variables, showing the amount of shared information between variable pairs. (**b**) Directional uncertainty coefficients (*U*(*X*|*Y*) and *U*(*Y*|*X*)), indicating the proportion of uncertainty in one variable that is reduced by knowing the other (see the ‘Methods’ section). The bar plots compare bacteriophage genera and species with three host-associated features: geographic origin (country), *Enterobacter* species (host) and isolation source. Higher mutual information and uncertainty coefficient values indicate stronger, non-random associations between phages and host features.

Although many clades included prophages from diverse geographic regions – suggesting broad global distribution – some clusters exhibited a moderate enrichment of prophages from specific countries, indicating possible regional circulation or localized transmission patterns. To evaluate whether prophage diversity is associated primarily with host species or geographic origin, we calculated the UC between all phage species and genera with respect to host species and country of origin (Fig. 3). We found that the residual uncertainty was 90.94% (U=0.90) considering phage species after knowing the country and 75.83% for the phage genera. Conversely, the residual uncertainty for the country after knowing the phage genus and species was 29.97 and 32.77%, respectively (Fig. 3). In other words, residual uncertainty determines how much information about a variable (X) you can obtain if you know another variable (Y). In this case, the UC is higher when species or genus is conditioned on country than the other way around, suggesting that geographic origin has a notable influence on phage lineage at both genus and species levels. Furthermore, phage species provide more information about geographic origin than phage genera, and vice versa.

It is important to note that this analysis was initially performed on the complete dataset of 3,778 phages, suggesting that singleton sequences may have strongly influenced the results. However, when restricting the analysis to genera with more than 10 members, the UC decreased to 77.61 and 75.88% when evaluating phage species in relation to country and host species, respectively (Table S3). Next, when analysing whether *Enterobacter* species were associated with geographic location and isolation source, we found that the uncertainty dropped to 13.05 and 9.41%, respectively (Table S3). These results indicate a clearer geographic structuring of phage populations, but not of *Enterobacter* species.

Finally, we were able to assign a taxonomic classification at the genus level to only 3.46% of our dataset, using ICTV reference genomes (see the ‘Methods’ section). Among these, only 36 prophages could be classified, with *Senquatrovirus* and *Novemvirus* being the most prevalent genera (Table S4). Moreover, all of them correspond to new phage species.

### Prophage species richness

We next assessed prophage species richness in relation to both bacterial host species and sources of isolation. Specifically, we quantified the diversity of prophages (prophage species) identified within each *Enterobacter* species, estimating how many distinct prophage species were associated with each host. Because most bacterial genomes in our dataset come from human-associated sources, and the majority were derived from *E. hormaechei*, we performed rarefaction analyses to compare prophage richness across *Enterobacter* host species and isolation sources, while controlling for differences in sampling effort.

**Fig. 4.**
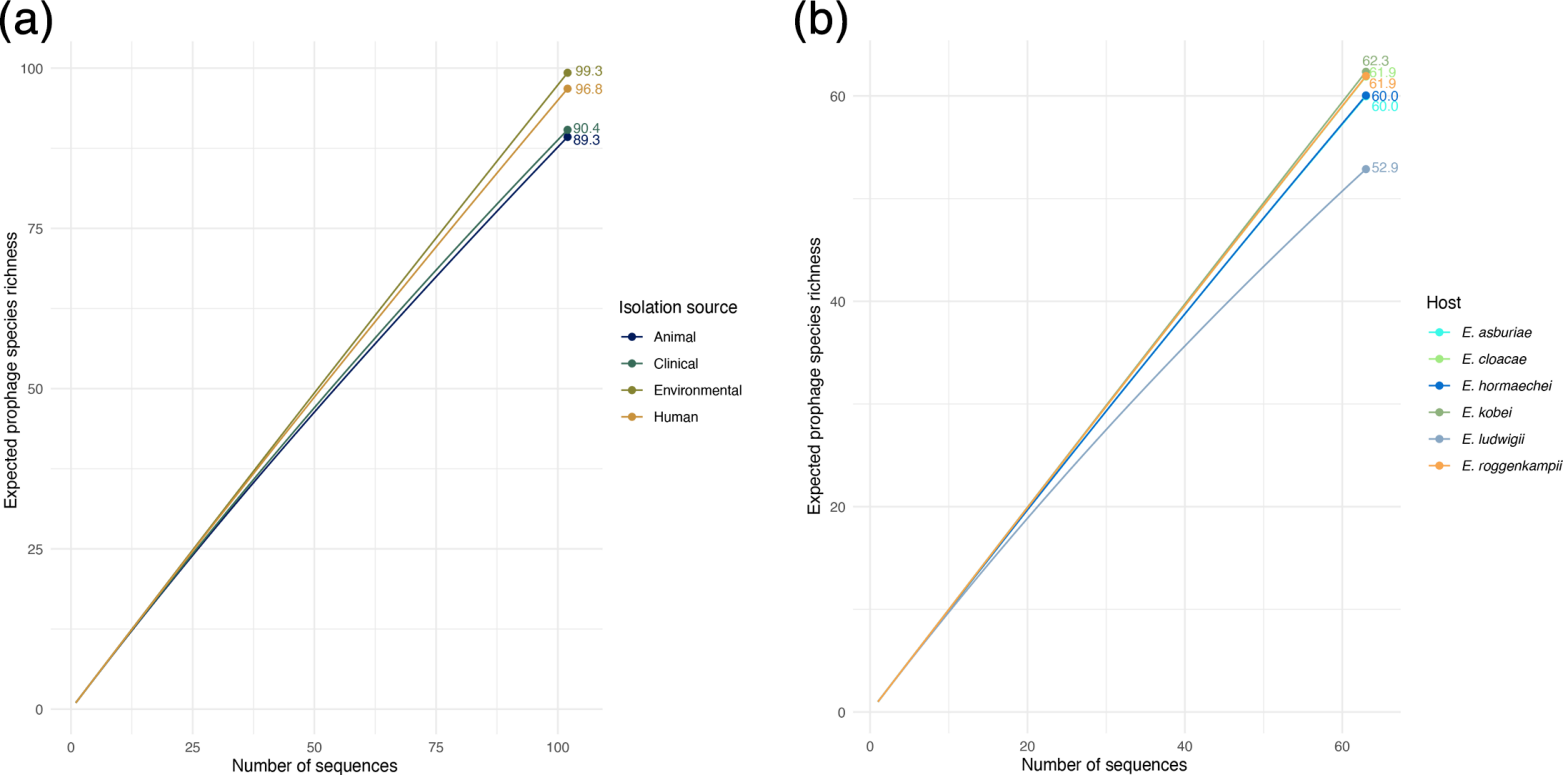
Rarefaction analysis of prophage species richness. Subsampling-based rarefaction curves were generated to estimate the expected richness of prophage species across different isolation sources (**a**) and *Enterobacter* species (**b**), while controlling for differences in sampling effort. Rarefaction for isolation sources was standardized, corresponding to 75% of the total sequences from the least sampled group (see the ‘Methods’ section).

For the analysis by isolation source, rarefaction was standardized to 102 sequences, representing 75% of the prophage count in the least sampled group (clinical). Under this standardized effort, environmental and human-associated sources exhibited the highest expected richness (99.3 and 96.8 prophage species, respectively), while animal and clinical sources had lower values (90.4 and 89.3 prophage species, respectively, Fig. 4a). Regarding host species, we included only those with at least 50 total prophage sequences and rarefied richness to 63 sequences, which corresponds to 75% of the prophage count from the least sampled host (*E. ludwigii*). Under these conditions, *E. kobei* displayed the highest expected richness (62.3 prophage species), followed by *E. cloacae* and *E. roggenkampii* (61.9 each), and then *E. asburiae* and *E. hormaechei* (60 each). *E. ludwigii* showed the lowest richness (52.9 prophage species) (Fig. 4b).

Rarefaction curves indicated a continuous accumulation of prophage species with increasing sampling effort across host species and isolation sources. These findings indicate that prophage diversity varies substantially depending on the bacterial host and ecological context, even after accounting for differences in sequencing depth. Notably, environmental and human-associated isolates, as well as certain *Enterobacter* species, harbour more diverse prophage populations.

### Coevolution and host switches inferred by PACo analysis

**Fig. 5.**
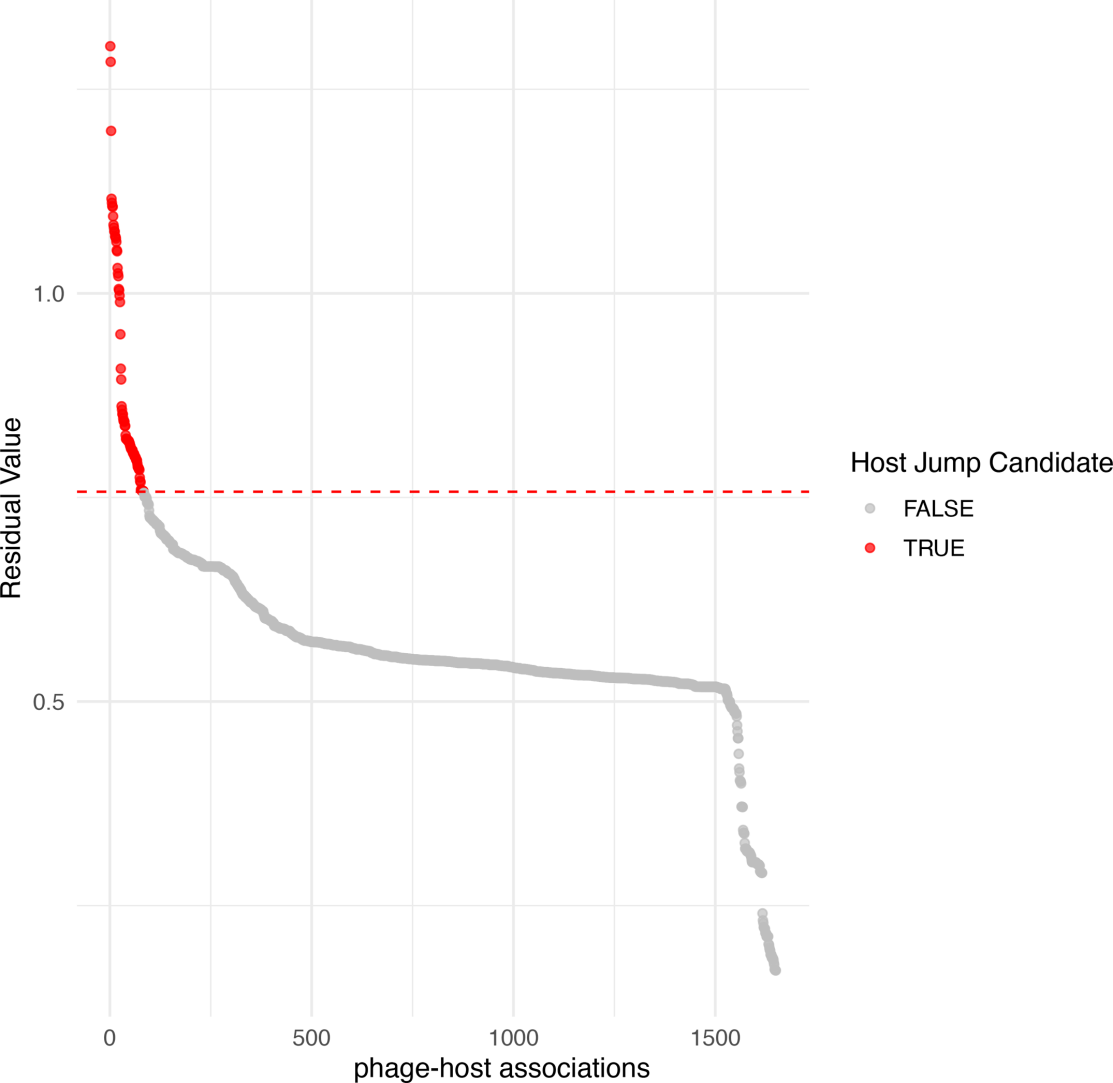
Plot of PACo residuals identifying candidate host jumps. Each point represents a unique association between an *Enterobacter* host and a prophage. Residual values (*Y*-axis) quantify the deviation of each association from the expected codivergence model, as inferred by PACo analysis (see the ‘Methods’ section). The *X*-axis ranks associations by increasing residual magnitude. Associations with residual values above the 95th percentile threshold (0.756; dashed red line) are highlighted in red and classified as potential host jump events (*n*=83, 5.03%), while the remaining associations (*n*=1,567, 94.97%) are consistent with codivergence.

Cophylogeny arises when two sets of phylogenies and their interactions are congruent, which may indicate a shared evolutionary history [55]. To assess the cophylogenetic signal between *Enterobacter* isolates and their prophages, we performed a PACo analysis using phylogenetic distances and host–phage association data (see the ‘Methods’ section). The analysis revealed a significant global fit, resulting in a *P*-value of 0 after 10,000 permutations, indicating dependence of prophage phylogeny on host phylogeny. Additionally, the global test yielded a significant ParaFitGlobal (*P*-value of 0.001, 999 permutations), confirming a strong overall cophylogenetic signal between *Enterobacter* hosts and their prophages. These results are consistent with the observed host specificity in most prophage clusters and the limited number of cross-species transmission events (Fig. 2b). We further investigated individual host–phage associations by calculating squared residuals, which quantify the deviation from the expected codivergence under the PACo model (1650 associations). To identify potential host-switching events, we classified associations with residuals above the 95th percentile (residual >0.756) as candidate host jumps. Based on this threshold, we identified 83 potential host jumps (5.03%) and 1567 associations consistent with codivergence (94.97%) (Fig. 5).

In other words, residuals represent deviations between the phylogenetic distances of hosts and their prophages. Thus, high residuals indicate that the current association between a phage and its host differs substantially from what would be expected under a strict co-divergence scenario. We observed that 40.96% (34 phages) of the candidate host jump events corresponded to phages belonging to cluster genus 6 (Fig. 2a), all identified in *E. ludwigii*. The remaining host-jump events involved prophages infecting seven different *Enterobacter* species (Fig. S5). The Procrustean superimposition plot (Fig. S5) displays the differences between prophage and host coordinates of patristic distances. We observed that hosts were grouped according to their species. This result aligns with the phylogenetic tree (Fig. 1a), whereas some of their prophages were located distantly. Notably, prophages classified as host jumps show longer lengths of the line (red lines), showing less prophage–host congruence (Fig. S5).

### Identification of defence and anti-defence systems

**Fig. 6.**
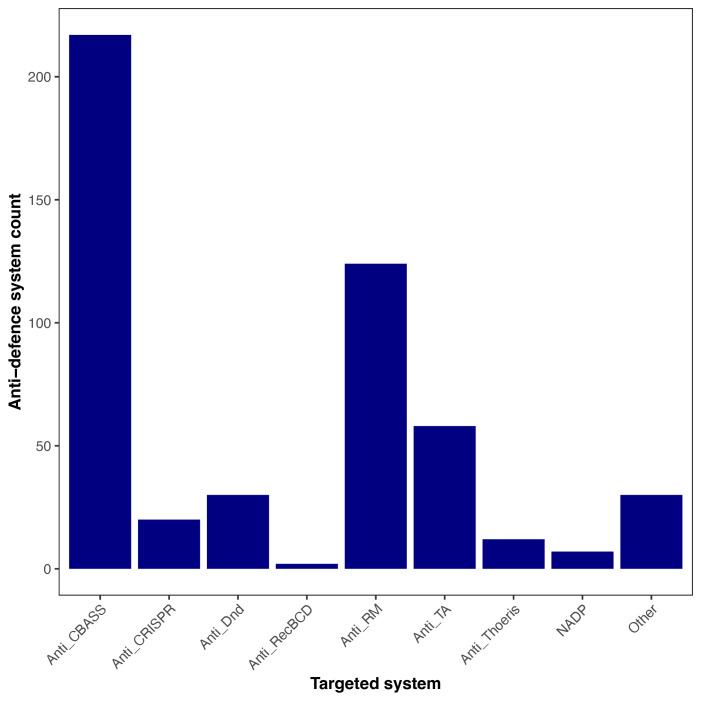
Anti-defence systems identified with AntiDefenseFinder in phage genomes. Total number of anti-defence systems detected across phage sequences that inhibit a specific type or family of defence systems.

The coevolution between prokaryotes and phages drives the diversification of both defence and anti-defence systems [61]. To explore these systems, we evaluated the presence of defence and anti-defence mechanisms in bacterial hosts and prophages, respectively (see the ‘Methods’ section). We found that the most prevalent defence system was a Phage Defense Candidate (PDC-S07), present in 99.06% of genomes (740 genomes), followed by RM_type_I, RM_type_II and gabija systems (Fig. S6). In contrast, only 8.86% of prophages encoded at least one anti-defence system. Of these systems, anti-CBASS and anti-RM were the most prevalent, representing 43.4% and 24.8% of detections, respectively (Fig. 6). Notably, RM systems (particularly types I and II) were the most abundant among the identified defence systems. Consistent with our findings, previous studies have reported that anti-CBASS and anti-RM are the most common anti-defence systems in *Enterobacter* phages [15]. We additionally screened all prophage genomes for ARGs using the RGI model implemented in CARD (based on protein homology and SNP detection). No prophage sequences met the criteria for bona fide ARGs using this approach. Although Pharokka detected a small number of putative virulence-associated genes (Table S5), these annotations require further validation.

## Discussion

Our analysis of prophage presence across *Enterobacter* species reveals their frequent occurrence and high diversity, providing a comprehensive catalogue that offers new insights into host–phage dynamics. This finding underscores the important role of prophages within these species and provides a framework for future studies to understand their evolutionary implications.

A recent large-scale study analysing over 13,000 bacterial genomes found that certain pathogens were enriched in prophage content. Notably, species within the genera *Acinetobacter*, *Pseudomonas* and *Enterobacter* showed significant prophage enrichment [17]. Our findings are consistent with this observation. In this study, we observed that almost all *Enterobacter* genomes analysed (98.92%) contained prophages and often harboured many different prophages. Furthermore, the high abundance of prophages corresponded to different phage lineages, showing a wide diversity of prophages at the species and genus levels. Another study analysing complete genomes of 11,827 plasmids and 2,502 phages from the NCBI non-redundant database identified a large number of ARGs encoded within mobile elements [62]. However, most predicted ARGs in phages turn out to be non-functional [63]. Additional studies focused specifically on *Enterobacter* species have also reported prophages carrying virulence or antibiotic resistance genes, although most of these analyses have been limited to a small number of *E. hormaechei* genomes associated with outbreaks [29].

Although some work has investigated prophages in a few *Enterobacter* species, no study has characterized prophage populations across the genus as comprehensively as in this work. By analysing 3,778 high-quality prophage sequences from diverse host species and ecological contexts, we provide the first genus-wide view of prophage diversity in *Enterobacter*. One of our main findings is the discovery of a previously unreported high prophage diversity. Specifically, we identified 1617 phage genera and 2,423 species, of which only 25 genera were recognized by the ICTV. Additionally, 79.98% of prophages were singletons (1,938 in total), suggesting that many more phage genera and species remain to be discovered. A potential limitation of our study is the overrepresentation of *E. hormaechei* genomes, mostly derived from human sources. However, the density of singletons appeared comparable across several *Enterobacter* species. For reference, singleton frequencies in other pathogens such as *Acinetobacter baumannii* have been reported to reach ~70% [19,21]. These results reveal a vast diversity of prophages harboured by *Enterobacter* species, a pattern that also seems to occur in *A. baumannii* [19,21]. Moreover, our data indicate that prophage species diversity remains largely unexplored, suggesting that broader and deeper sampling could uncover even greater diversity. Notably, some human-associated pathogenic species such as *E. kobei* and *E. cloacae*, as well as *E. roggenkampii* – a pathogen reported in plants, animals and humans – exhibited high prophage species diversity. Similarly, isolates from environmental and human-associated sources showed the highest expected prophage species richness. These results illustrate the concept of ‘viral dark matter’ [64], which refers to viral species that have not been characterized but whose existence has been revealed through metagenomic data and, in this case, by exploring host genomes. In contrast, prophage populations in other pathogens, such as *Streptococcus agalactiae* and *Campylobacter* species, appear to be less diverse [65,66]. On the other hand, the lack of reports on inducible prophages in *Enterobacter*, in contrast to other pathogens such as *A. baumannii* [19], highlights how little is known about the induction potential of prophages in this genus. Our findings revealed a high diversity of prophages harboured in *Enterobacter* species. This underscores the need for complementary experimental approaches to determine which prophages remain active in the host.

From an ecological perspective, it is important to understand which phage species are circulating within specific geographic regions, hosts and isolation sources. The uncertainty coefficient revealed that phage species exhibited strong associations with the geographic origin, host species and isolation source (*U*≥0.80). This pattern suggests that the diversity and distribution of *Enterobacter* phages are strongly influenced by host-related and environmental factors. Although this relationship was strongest when including singletons, the uncertainty coefficients remained high even when singletons were excluded from the analysis (see the ‘Methods’ section). In other words, phages tend to be species-specific within *Enterobacter*. Furthermore, geography seems to influence the distribution of phage species, but not necessarily genera, suggesting local selective pressures or coevolutionary dynamics. Although our dataset is enriched for *E. hormaechei* isolates (~60% of the hosts), these genomes originate from diverse countries and isolation sources, minimizing the potential impact of sampling bias. The broad geographic distribution (over 35 countries, with no single country exceeding 22% of isolates). Moreover, the uncertainty coefficient (*U*≥0.80) supports this association, and the observed phage–host cophylogeny reflects evolutionary patterns rather than sampling bias. In this sense, another important result is that host–prophage associations were not random and appear to be shaped by codiversification or host-specific ecological constraints. Our findings indicate that while most associations show strong phylogenetic congruence – suggesting long-term coevolution – there is also evidence of recent or past host-switching events involving a small subset of prophages, pointing to possible regional circulation or localized transmission patterns. Although this approach excludes a large proportion of singleton prophages, it ensures a more stable phylogenetic structure suitable for cophylogenetic analyses. This may bias the results toward more conserved or widespread phage lineages, but allows for statistically meaningful comparisons.

It should be noted that prophages from cluster one were found in six different species of *Enterobacter* from different countries, which was an atypical event. Exchange of prophages among isolates from different sources (e.g. environmental vs. clinical), even within the same host species, was rare, further indicating that prophage transmission may be constrained by ecological interactions or niche-specific adaptations. Collectively, these findings suggest that while prophages may occasionally cross host species boundaries, such events are infrequent and likely driven by specific biological or ecological circumstances. Therefore, circulating strains within distinct regions or ecological niches may influence susceptibility or resistance to certain phages.

From a public health perspective, it is also noteworthy that *β*-lactamase genes were more frequently associated with human (hospital-acquired) *Enterobacter* isolates [67]. While plasmids have been more extensively studied in this regard [62], recent evidence also implicates phages [29]. In this study, we did not detect clear evidence of ARGs in our dataset using the Resistance Gene Identifier model (based on protein homology and SNPs). This absence may be attributed to our stringent criteria for selecting bona fide prophages, which ensured that only complete prophage genomes were retained. Although multiple studies have reported prophage-encoded ARGs, these are often found in degraded or incomplete prophages [7,68]. Although Pharokka identified 166 putative virulence genes, mostly involved in immune modulation, across 166 prophages, further analyses (similar to the RGI model) are required to confirm these findings.

Thus, considering all our results together, we propose two hypothetical scenarios regarding the potential impact and mobility of prophage within *Enterobacter* populations.

First, if these prophages become activated and propagate within host populations, they are likely to transfer such advantages (if they contain virulence genes or ARGs) only to very closely related hosts. Our cophylogeny results suggest that this restriction could be mediated by coevolution between phages and hosts. Geographic barriers and/or host lifestyle may further restrict this dissemination. This is likely because the majority of prophage species were associated with the isolation source of their respective bacterial hosts. In other words, prophage-mediated spread of ARGs or VGs across *Enterobacter* species is likely limited. Second, most *Enterobacter* prophages appear to be undergoing a process of domestication and may be doomed to persist passively within their host genomes, since domesticated prophages are under purifying selection [11]. Following this idea, our results suggest that only ‘host jumps’ candidates (see Fig. 4) might remain active (5.03%). Although our analysis revealed phages of the same species infecting different host species (as shown by the network in Fig. 2b), these observations suggest that some prophages may have a broader host range. However, further analyses combined with experimental evidence are needed to identify active prophages. From this perspective, the widespread presence of prophage signatures could serve more as a barrier against novel phage infections, where resident prophages promote the formation of defective particles [11].

Finally, among *Enterobacter* genomes, PDC-S07, RM type I-II and gabija systems predominated, reflecting a strong selective pressure to maintain antiviral protection against bacteriophages. In contrast, only a small fraction of prophages encoded anti-defence systems, mainly anti-CBASS and anti-RM systems. Consistent with large-scale surveys reporting uneven distributions of anti-defence systems among phages [15], only 8.9% of *Enterobacter* prophages encoded such mechanisms, predominantly anti-CBASS and anti-RM. This lower proportion suggests different evolutionary pressures acting on integrated prophages (phage domestication), which can accumulate mutations or lose accessory functions over time [11].

Overall, our study provides a comprehensive view of prophage diversity, distribution and host associations within *Enterobacter* species, revealing how evolutionary and ecological forces shape the prophage population. The observed host specificity, coevolutionary patterns and diversity of defence and anti-defence systems highlight the complexity of phage–host interactions in this genus. These findings emphasize the need to consider both host–phage evolutionary relationships and local ecological contexts when designing phage therapy strategies, particularly against multidrug-resistant *Enterobacter* strains.

## Supplementary material

10.1099/mic.0.001660 Uncited Supplementary Material 1.

10.1099/mic.0.001660 Uncited Supplementary Material 2.

## Abbreviations

NCBI: National Center for Biotechnology Information
