# Supplementary material for: Extensive hidden prophage diversity in Enterobacter species reveals host specificity and local distribution
Source: Microbiology (Reading). 2026 Jan 28;172(1):001660. doi: 10.1099/mic.0.001660 (PMC12851794; doi:10.1099/mic.0.001660)
Supplement: Uncited Supplementary Material 1. [file mic-172-01660-s001.pdf]

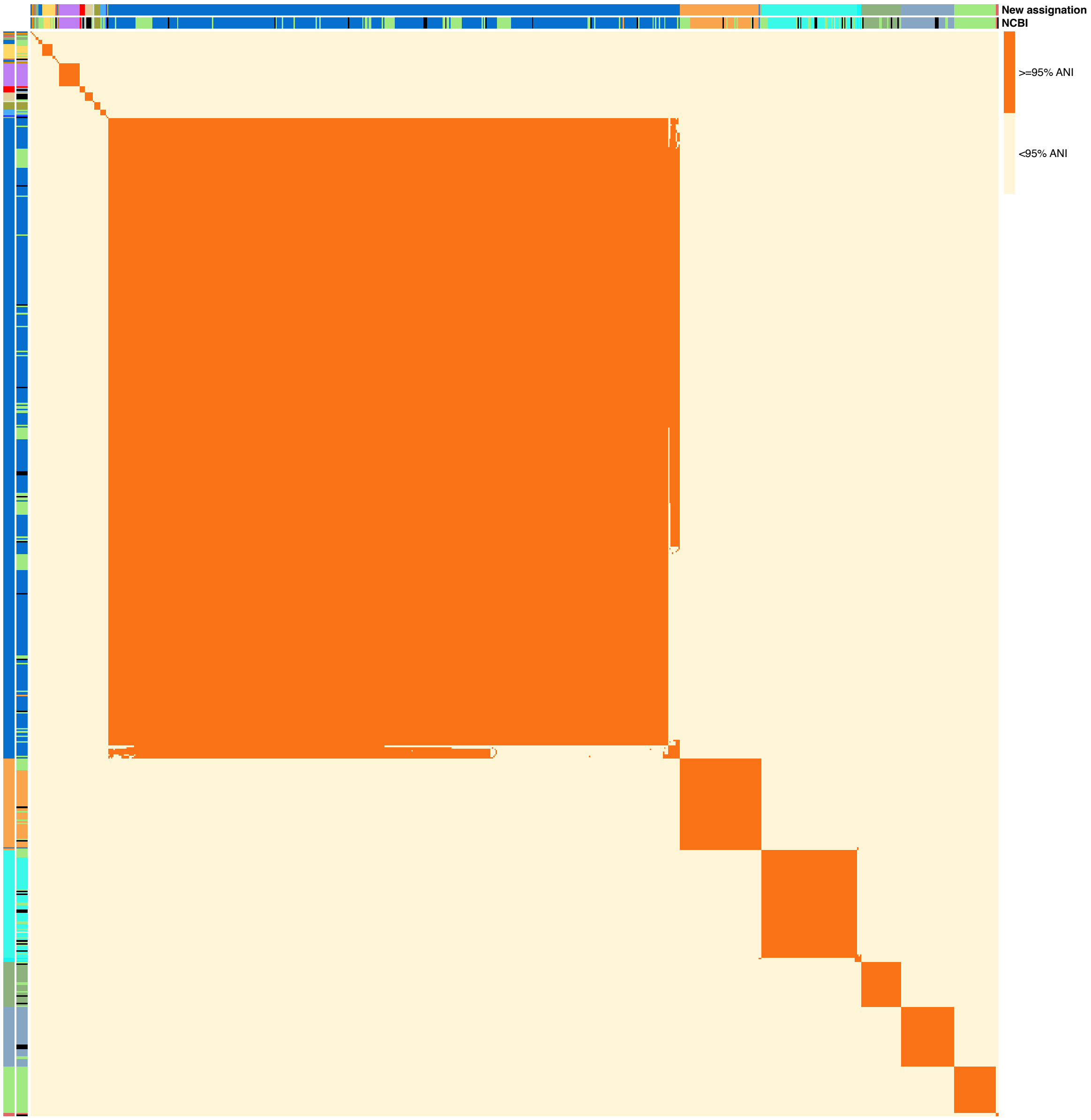

Supplementary Figure S1. Heatmap of average nucleotide identity (ANI) among 747 *Enterobacter* genomes. Orange cells represent values equal to or greater than 95% identity, indicating that the corresponding genomes belong to the same species, whereas yellow cells represent values below this threshold. The top row indicates species classifications according to NCBI taxonomy and ML-based phylogeny.

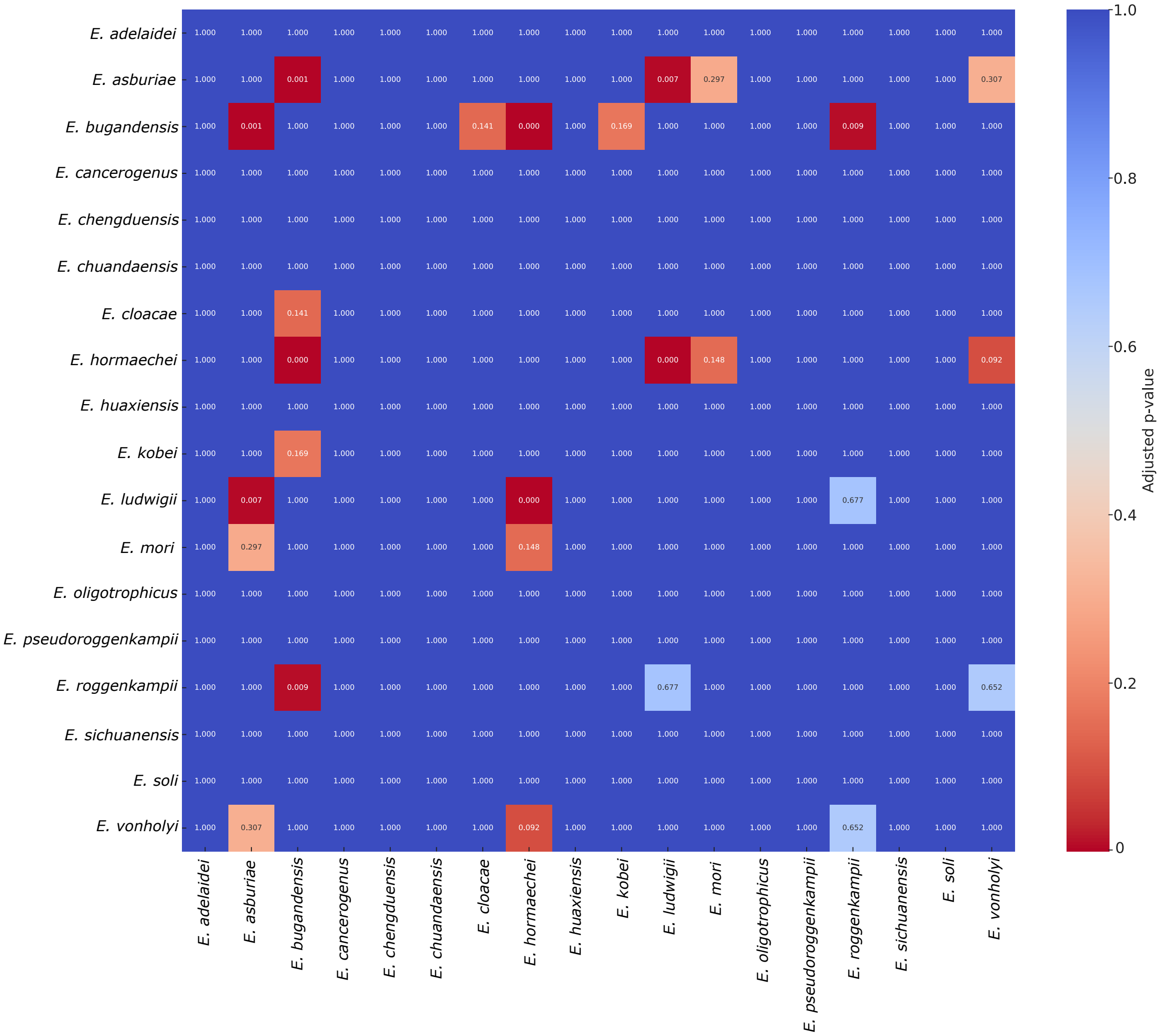

Supplementary Figure S2. Pairwise comparisons of prophage abundance across *Enterobacter* species. Pairwise statistical differences were evaluated using the Dunn test following a significant Kruskal–Wallis result ($H = 89.44$, $p < 0.0001$). The heatmap displays adjusted *p*-values from Holm-Bonferroni-corrected comparisons between *Enterobacter* species. Color intensity reflects statistical significance, with red indicating lower p-values (more significant differences) and blue indicating higher p-values (non-significant comparisons). Species are ordered alphabetically. Comparisons with adjusted $p \geq 0.05$ are considered non-significant.

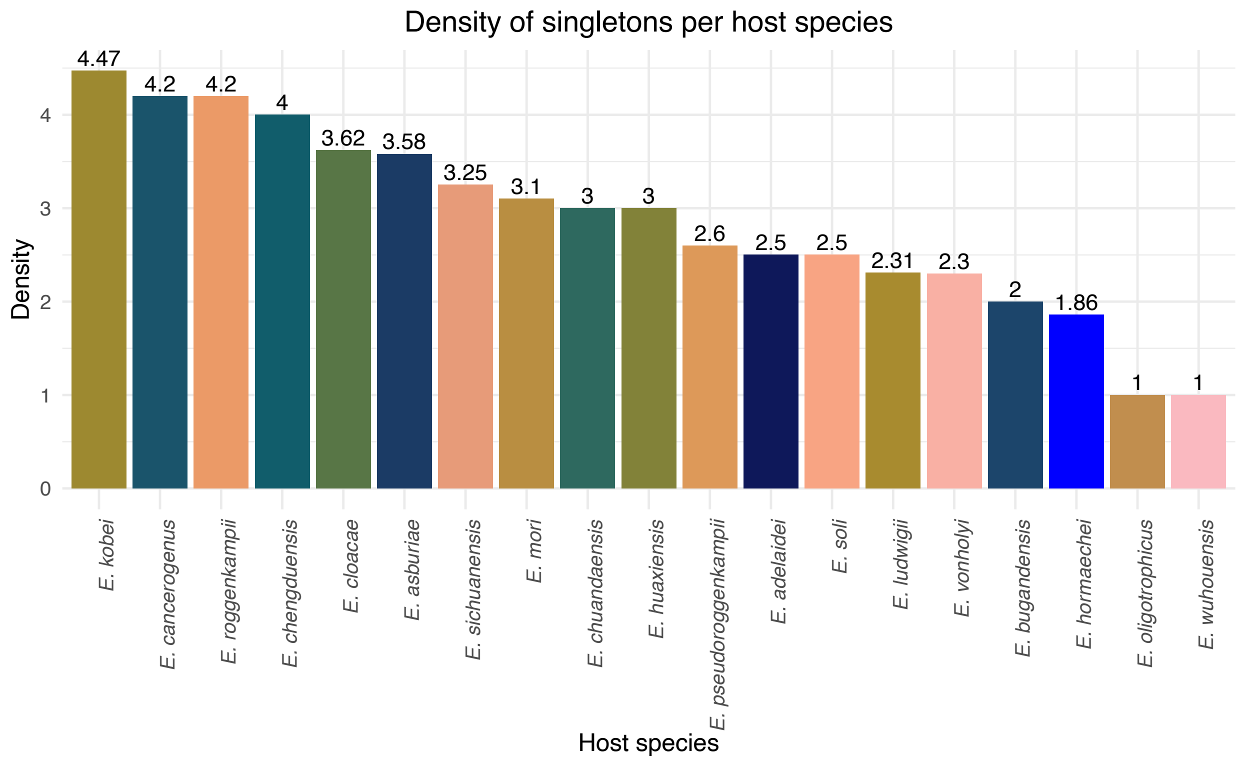

Supplementary Figure S3. Density of singleton prophage species per *Enterobacter* host. The barplot shows the density of singleton prophages identified in each *Enterobacter* species.

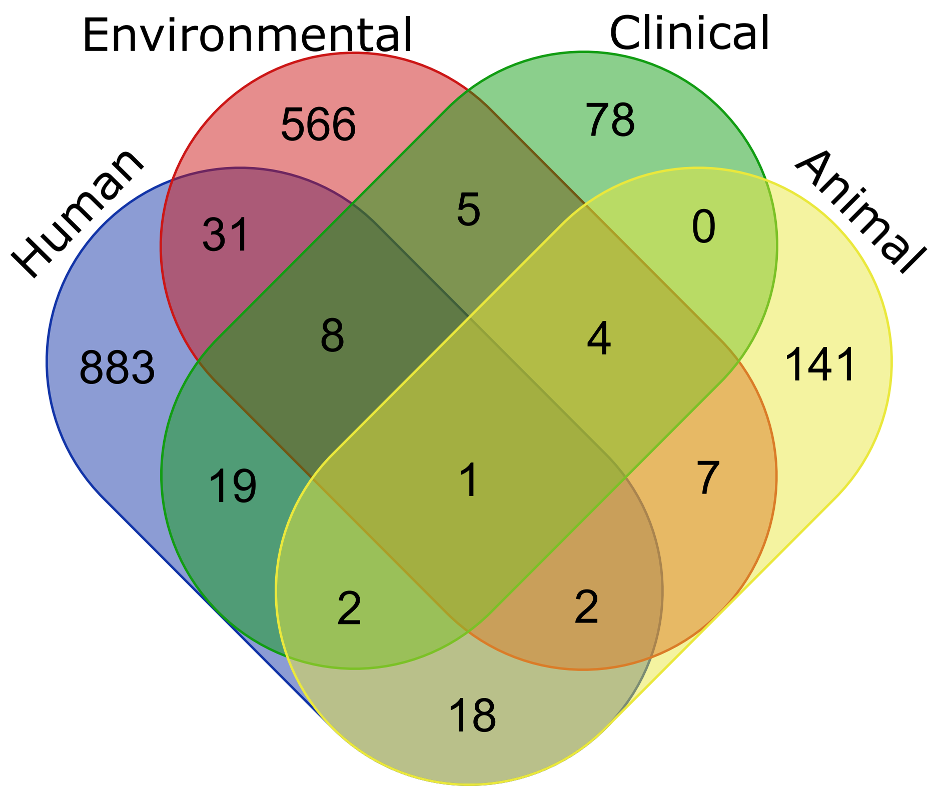

Supplementary Figure S4. Venn diagram of phage species found in *Enterobacter* genomes across different isolation sources. The diagram shows the overlap of prophage species identified in *Enterobacter* isolates from four distinct isolation sources: Clinical, Human, Environmental, and Animal (see Materials and Methods). Each area represents the set of unique phage species found in that category, with intersections indicating shared prophage species across sources.

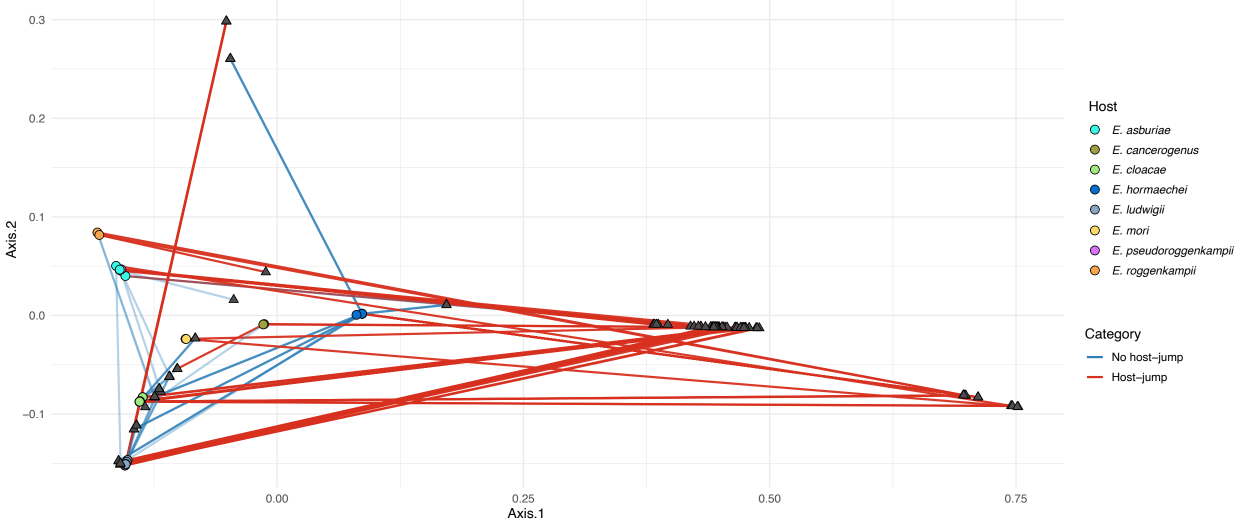

Supplementary Figure S5. The Procrustean superimposition plot shows the association between prophages and their *Enterobacter* hosts, that harbors a host-jump candidates. The analysis was performed using PACo (see Materials and Methods). Circles represent hosts, colored by *Enterobacter* species, and triangles represent prophages. Lines indicate the residual distances between host and prophage coordinates derived from patristic distances. Red lines correspond to prophages inferred to have undergone host jumps, whereas blue lines represent associations consistent with codivergence. Longer lines indicate lower congruence between host and prophage phylogenies. The projection of residuals onto the first two axes is given by the length of the line.

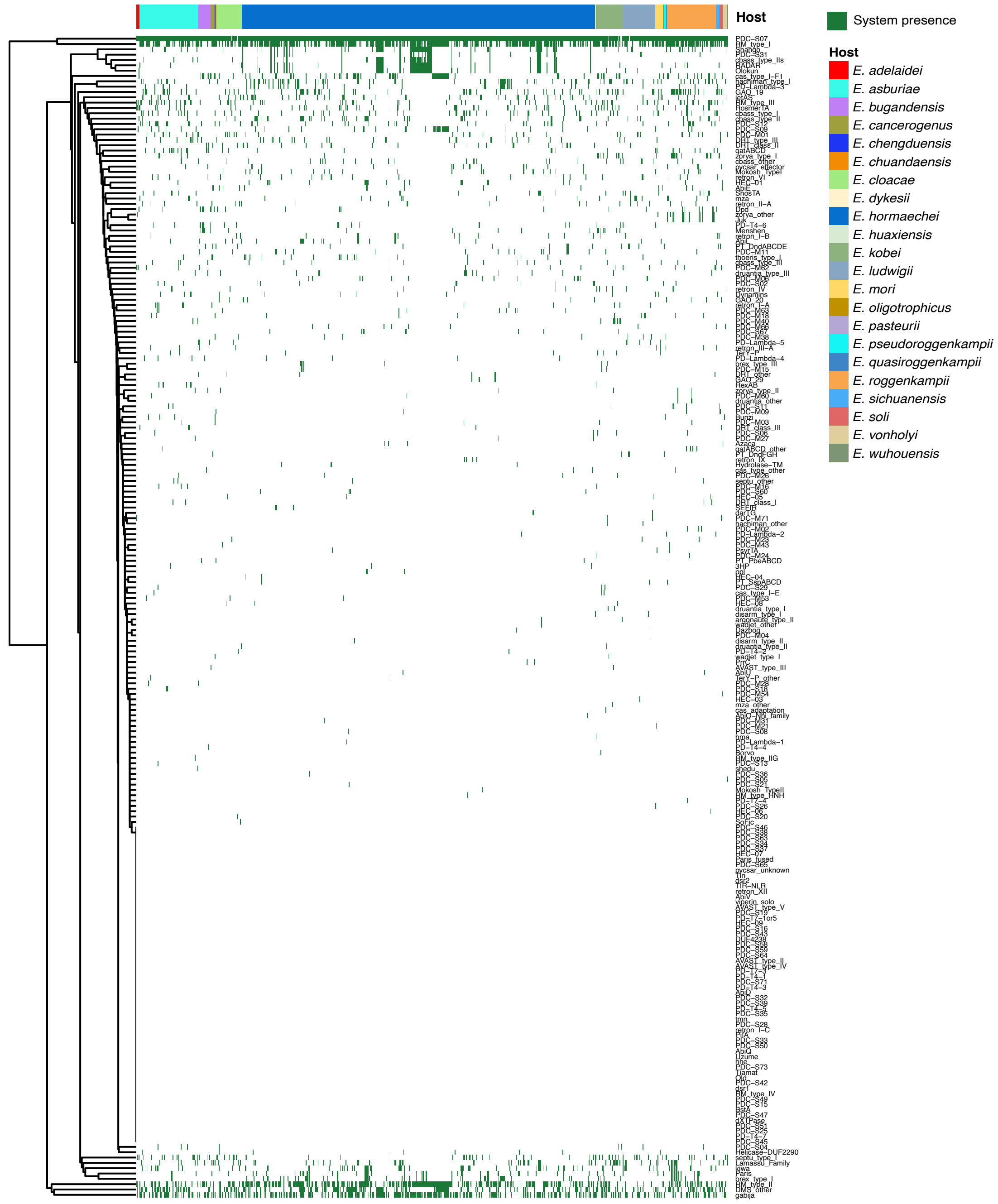

Supplementary Figure S6. Heatmap of defense systems detected in 747 *Enterobacter* genomes. Defense systems were identified using PADLOC, and a system was considered present according to previous report (42), or when at least two component proteins were detected within the same genome. Rows represent defense system types, and columns correspond to *Enterobacter* genomes, color-coded by host species. The green cells indicate system presence, while white cells indicate absence. The hierarchical clustering of genomes (top dendrogram) reflects the overall similarity in defense system profiles among species.
